# Supplementary material for: Dual-energy three-compartment breast imaging for compositional biomarkers to improve detection of malignant lesions
Source: Commun Med (Lond). 2021 Aug 31;1:29. doi: 10.1038/s43856-021-00024-0 (PMC9053198; doi:10.1038/s43856-021-00024-0)
Supplement: Supplementary file 2 — Supplementary Information [file 43856_2021_24_MOESM2_ESM.pdf]

SUPPLEMENTARY METHODS

3CB Imaging

All images were acquired from women scheduled for percutaneous breast biopsy, prior to their biopsy. We used a single Hologic Selenia full-field digital mammography system (Hologic, Inc. Bedford, MA) to image women with 3CB. This particular system configuration has a molybdenum X-ray anode and two internal X-ray filters of either

Molybdenum or Rhodium. Two mammograms were acquired on each woman's affected breast using a single compression. The first exposure was made to mimic the clinical screening mammogram conditions such that the Selenia's internal software chooses the voltage and current settings based on breast thickness usually below 30 kVp. The second mammogram was acquired at a fixed voltage (39 kVp) and current for all participants. An additional 3-mm thick X-ray filter was placed in the beam path to remove more of the low energy X-rays. A high energy exposure (39 kVp/Rh filter) was made using an additional 3-mm plate of copper in the beam to increase the average energy of the high energy image. The 39 kVp high-energy voltage is the highest obtainable voltage on the Selenia unit. We limited the total dose of this procedure to be approximately 110% of the mean-glandular dose of an average screening mammogram. The images were collected under an investigational review board approval to measure breast composition. The calibration standards and 3CB algorithms for generating compositional thickness maps have been previously described in full[1,2].

Lesion Diagnosis

Pathology was reported on all biopsied lesions at both clinical sites (University of California, San Francisco, San Francisco, Calif, and H. Lee Moffitt Cancer Center, Tampa, Fla) to determine ground truth diagnosis. Hormone receptor assays were performed on lesions with malignant pathologies to identify estrogen receptor, progesterone receptor, and human epithelial receptor 2 status.

Biopsy Site Delineations

Sub-specialized breast radiologists at both clinical sites used pre-biopsy mammograms and pathology reports to delineate the site of the biopsy. Custom software was used to capture the delineations and store the x and y coordinates of each delineation point. Medical grade, high resolution radiology monitors were used for viewing and delineations. Presentation images used for radiologist readings and delineations are co-registered with the resulting 3CB thickness maps. Therefore, the delineation coordinates will be projected in the exact location on all 3CB thickness maps.

CAD Delineations

Patients' diagnostic mammograms, in raw DICOM format, were pushed to a local iCAD PowerLook server running CAD software. Using iCADs CAD proprietary algorithm at the most sensitive setting, the iCAD delineate suspicious masses and each individual calcification within a calcification cluster. CAD also assigned a probability of malignancy for each suspicious mass and calcification cluster. The x and y coordinates of each CAD delineation was captured and stored in an output Extensible Markup Language (XML) file.

Some patients present with numerous calcifications and the total number of individual calcifications significantly outnumber the total number of masses identified by CAD. In order to address this possible imbalance, new delineations were generated which delineated an ROI for a calcification cluster rather than use the delineation for all individual calcifications. CAD automatically indexed and grouped each calcification. The new ROI was calculated by taking all calcification within a cluster, extracting the set of x and y coordinates, and calculating the convex hull for that set of all coordinates[3,4].

3CB Feature Engineering

Three outer region ROIs were generated around the lesion ROI to evaluate the area and tissue immediately surrounding a lesion. Each outer region ROI was 2mm in thickness and thus 2mm away from the border of the previous ROI. Using the lesion ROI coordinates, a Euclidean distance transform[4] was used to compute coordinates 2mm away from the lesion border, for the first outer region. This same method was used to compute the second outer region and third outer region by using the first outer region and second outer

region as the reference points for the Euclidean distance transform respectively. As a result, each lesion has a total of four ROIs; one for the lesion and three outer regions 2mm in thickness at distances of 2, 4, and 6mm from the lesion ROI, see Figure 2b.

Single numeric values, which includes mean, median, standard deviation, minimum, maximum, kurtosis, skew, total and percentage, were captured to characterize each ROI. These nine values were calculated for all four lesion ROIs on each of the three 3CB thickness maps. A total of 108 total compositional features were extracted from every single lesion.

Data Augmentation

To combat overfitting of our model, data augmentation was implemented at the level of ROI delineations. The 3CB thickness maps contain the point thicknesses of a given composition (LWP) on a pixel by pixel basis and the 108 extracted features are derived from all pixels within an ROI. It is unlikely that an ROI delineation perfectly captures all pixels corresponding to a lesion while perfectly excluding pixels corresponding to normal breast tissue. In addition, human variability explains that delineation ROIs for the same lesion will not result in the exact same coordinates despite delineations originating from the same radiologist. Therefore, our augmentation strategy was meant to account for possible variability involved with delineating ROIs. ROIs were rotated within a range 10 degree and translated in any direction within a range of five pixels. Rotation and translation augmentations were not mutually exclusive, and a new augmented ROI could have undergone both transformations. Features were extracted from all newly augmented ROIs and included into the training set. This augmentation was performed as a preprocessing step as opposed to during training. Augmentation ROIs only appeared in the training set and the unseen test set for which results are report contained no augmentations.

Modeling

Our final predictive model was constructed with the Python programming language using the Keras machine learning API. The neural network consisted of two main components. The first component consisted of five, 2048 node layers and it received the 108 extracted 3CB features from a lesion as input. The output from the first component was used as input for the second component which consisted of two,

512 node layers. In addition to the output from the first component, the second component also received CAD's predicted probability of malignancy as input. A single node prediction layer, at the end of the second component, output a predicted the probability of malignancy. The learning rate, decay, dropout, number of nodes, number of layers, and batch size were all optimized and determined using the Sherpa, a hyperparameter optimization tool, population based genetic algorithm.[5,6] The search spaces for each optimized hyperparameter are as follows: between 1E-8 and 1 for learning rate, between 1E-8 and 1 for decay, 0.1-0.9 for dropout, 128-2048 for number of nodes, 3-10 number of layers, and 2-512 for batch size. The hyperparameter combination which resulted in the best AUC on the validation set was used to train the final model. The best hyperparameters used in the final model are as follows: learning rate of 1.5E-5, decay of 5.18E-3, dropout of 0.2, 2048 nodes, 7 layers, and a batch size of 512. The final model was trained on the training set and the model was frozen. The unseen test set was evaluated by the trained, frozen model, and those results are reported in Figure 3.

Statistics

The Scikit Learn Python machine learning package was used to compute AUCs. The mean AUCs and 95% confidence intervals were computed via 1000 bootstrapping samples. The p-value significances in Figure 5 and Table 4. were calculated by Welch's test for unequal variance.

REFERENCES

1. Laidevant, A.D., Malkov, S., Flowers, C.I., Kerlikowske, K. & Shepherd, J.A. Compositional breast imaging using a dual-energy mammography protocol. *Med Phys* **37**, 164-174 (2010).
2. Malkov, S.*, et al.* Calibration procedure of three component mammographic breast imaging. in *International Workshop on Digital Mammography* 211-218 (Springer, 2016).
3. Graham, R.L. An efficient algorithm for determining the convex hull of a finite planar set. *Info. Pro. Lett.* **1**, 132-133 (1972).
4. Preparata, F.P. & Shamos, M.I. *Computational geometry: an introduction*, (Springer Science & Business Media, 2012).
5. Hertel, L., Collado, J., Sadowski, P. & Baldi, P. Sherpa: Hyperparameter Optimization for Machine Learning Models. (2018).
6. Jaderberg, M.*, et al.* Population based training of neural networks. *arXiv preprint arXiv:1711.09846* (2017).

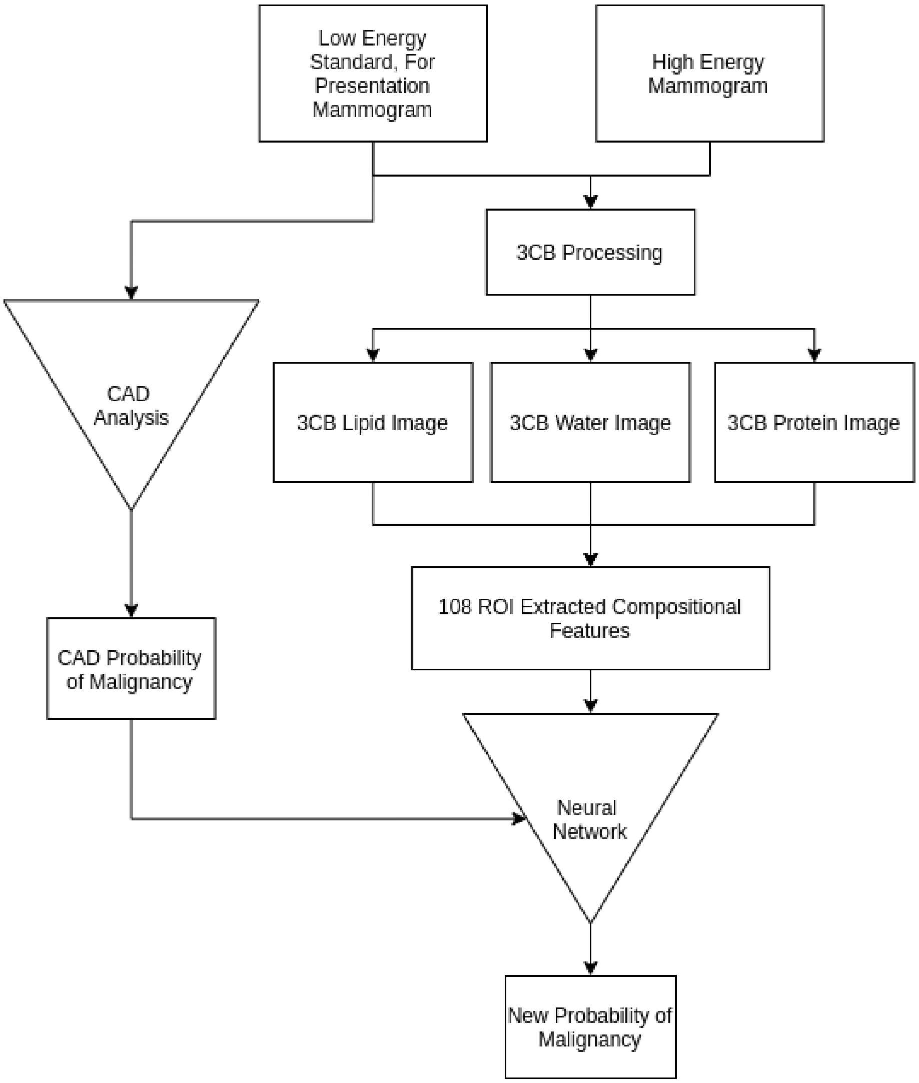

Supplemental Figure 1. Processing flow diagram. Low energy standard mammograms are processed by CAD to obtain a probability of malignancy. The high energy and low energy mammogram are used to produce 3CB compositional thickness maps, lipid, water, and protein. Features are extracted from the 3CB images and used as input into the neural network.
