# Supplementary material for: Dual-energy three-compartment breast imaging for compositional biomarkers to improve detection of malignant lesions
Source: Commun Med (Lond). 2021 Aug 31;1:29. doi: 10.1038/s43856-021-00024-0 (PMC9053198; doi:10.1038/s43856-021-00024-0)
Supplement: Supplementary file 4 — Description of Additional Supplementary Files [file 43856_2021_24_MOESM4_ESM.pdf]

Description of Additional Supplementary Files

File name: Supplementary Data 1

Description: Source data for Figure 3, Figure 5, Figure 6, Table 3, and Table 4. Malignancy labels, predictions by computer-aided detection and prediction by the neural network are contained in columns entitled path, cad_pred, and nn_pred, respectively. Positive estrogen receptor, progesterone receptor, or human epidermal growth factor receptor 2 are denoted as ER+, PR+, or HER2+, respectively.
