# Supplementary material for: Dual-energy three-compartment breast imaging for compositional biomarkers to improve detection of malignant lesions
Source: Commun Med (Lond). 2021 Aug 31;1:29. doi: 10.1038/s43856-021-00024-0 (PMC9053198; doi:10.1038/s43856-021-00024-0)
Supplement: Supplementary file 5 — Reporting Summary [file 43856_2021_24_MOESM5_ESM.pdf]

nature research

Corresponding author(s): John Shepherd
COMMSMED-21-0137A

Last updated by author(s): 07-22-2021

Reporting Summary

Nature Research wishes to improve the reproducibility of the work that we publish. This form provides structure for consistency and transparency in reporting. For further information on Nature Research policies, see our Editorial Policies and the Editorial Policy Checklist.

Statistics

For all statistical analyses, confirm that the following items are present in the figure legend, table legend, main text, or Methods section.

n/a	Confirmed	
☐	☒	The exact sample size (*n*) for each experimental group/condition, given as a discrete number and unit of measurement
☐	☒	A statement on whether measurements were taken from distinct samples or whether the same sample was measured repeatedly
☐	☒	The statistical test(s) used AND whether they are one- or two-sided<br>*Only common tests should be described solely by name; describe more complex techniques in the Methods section.*
☐	☒	A description of all covariates tested
☐	☒	A description of any assumptions or corrections, such as tests of normality and adjustment for multiple comparisons
☐	☒	A full description of the statistical parameters including central tendency (e.g. means) or other basic estimates (e.g. regression coefficient) AND variation (e.g. standard deviation) or associated estimates of uncertainty (e.g. confidence intervals)
☐	☒	For null hypothesis testing, the test statistic (e.g. F, t, r) with confidence intervals, effect sizes, degrees of freedom and P value noted<br>*Give P values as exact values whenever suitable.*
☒	☐	For Bayesian analysis, information on the choice of priors and Markov chain Monte Carlo settings
☒	☐	For hierarchical and complex designs, identification of the appropriate level for tests and full reporting of outcomes
☒	☐	Estimates of effect sizes (e.g. Cohen's *d*, Pearson's *r*), indicating how they were calculated

Our web collection on statistics for biologists contains articles on many of the points above.

Software and code

Policy information about availability of computer code

Data collection	The iCAD inc, 114 Nashua, NH, SecondLook (version 7.2) commercial computer-aided detection (CAD) software was used for this work.
Data analysis	Custom 3CB software (version 2020) for image analysis and lesion delineation, and trained model, 3CB-CNN, are available at https://github.com/shepherd-lab/3cb_software_and_model/tree/V0.1.1, DOI: 10.5281/zenodo.461542142.

For manuscripts utilizing custom algorithms or software that are central to the research but not yet described in published literature, software must be made available to editors and reviewers. We strongly encourage code deposition in a community repository (e.g. GitHub). See the Nature Research guidelines for submitting code & software for further information.

Data

Policy information about availability of data

All manuscripts must include a data availability statement. This statement should provide the following information, where applicable:

- Accession codes, unique identifiers, or web links for publicly available datasets
- A list of figures that have associated raw data
- A description of any restrictions on data availability

Source data for the main figures in the manuscript can be found in “Supplementary Data 1”. Imaging data linked to extensive meta data could be used to identify a participant. The data that support the findings of this study are available from the corresponding author upon reasonable request through a data sharing agreement.
Custom 3CB software (version 2020) for image analysis and lesion delineation, and trained model, 3CB-CNN, are available at https://github.com/shepherd-lab/3cb_software_and_model/tree/V0.1.1, DOI: 10.5281/zenodo.4615421.

Field-specific reporting

Please select the one below that is the best fit for your research. If you are not sure, read the appropriate sections before making your selection.

☒ Life sciences ☐ Behavioural & social sciences ☐ Ecological, evolutionary & environmental sciences

For a reference copy of the document with all sections, see nature.com/documents/nr-reporting-summary-flat.pdf

Life sciences study design

All studies must disclose on these points even when the disclosure is negative.

Sample size	Power and sample size calculations were performed prior to the submission of the grant from which the data for this manuscript reports on. The sample size used in this manuscript was determined by the ability to curate completed data sets on each participants. Participants were excluded form this study if they did not have a complete data set which includes mammogram images, pathology on biopsies, radiologist delineations of biopsy sites, and three compartment imaging.
Data exclusions	Data was exclude if a participant was missing any piece of data used in the analysis. This data includes a diagnostic mammogram, pathology of breast biopsy, radiologist delineations and coordinates of the biopsy site, and a complete three compartment breast (3CB) dataset. The 3CB data set contains lipid, water, and protein thickness maps that are fully registered to the diagnostic mammograms. The 3CB protocol requires calibration images of phantoms to be taken prior to patient image acquisition. If these calibration images were missing or the registration of compositional thickness maps to diagnostic mammograms were incorrect, the 3CB data set was considered invalid and that patient was excluded.
Replication	Machine learning models were validated on a hold out, unseen, test set. This mitigates overfitting and enables a level of reproducibility in that our final models are generalizable and our reported performance results are not inflated. Final models will also be made publicly available.
Randomization	The data was randomly split into a train, validation, and test set by patient ID. Splitting the data by patient ID was used to combat data leakage or the possibility that highly correlated data points from one patient does not end up in more than one sub data set. Random splitting of the data was also performed under the constraint that each sub dataset (train, validation, and test) contain equal proportions of cancer and benign patients. Therefore the distribution of outcomes in each subset is representative of the overall study population.
Blinding	Blinding was not applicable to this study

Reporting for specific materials, systems and methods

We require information from authors about some types of materials, experimental systems and methods used in many studies. Here, indicate whether each material, system or method listed is relevant to your study. If you are not sure if a list item applies to your research, read the appropriate section before selecting a response.

Materials & experimental systems

n/a	Involved in the study	
☒	☐	Antibodies
☒	☐	Eukaryotic cell lines
☒	☐	Palaeontology and archaeology
☒	☐	Animals and other organisms
☐	☒	Human research participants
☐	☒	Clinical data
☒	☐	Dual use research of concern

Methods

n/a	Involved in the study	
☒	☐	ChIP-seq
☒	☐	Flow cytometry
☒	☐	MRI-based neuroimaging

Human research participants

Policy information about studies involving human research participants

Population characteristics	Women who were scheduled for breast biopsy for suspicious lesions (Breast Imaging-Reporting and Data System (BI-RADS) diagnostic categories 4 and 5) were recruited at the University of California San Francisco (San Francisco, CA) and H. Lee Moffitt Cancer Center and Research Institute (Tampa, FL).
Recruitment	Sequential women
Ethics oversight	Committee of human research, University of California San Francisco (San Francisco, CA) and H. Lee Moffitt Cancer Center and Research Institute (Tampa, FL).

Note that full information on the approval of the study protocol must also be provided in the manuscript.

Clinical data

Policy information about clinical studies

All manuscripts should comply with the ICMJE guidelines for publication of clinical research and a completed CONSORT checklist must be included with all submissions.

Clinical trial registration	Not applicable/Not a clinical trial
Study protocol	Not applicable/Not a clinical trial
Data collection	Not applicable/Not a clinical trial
Outcomes	Not applicable/Not a clinical trial
